# Effect of Thermal Processing on Physico-Chemical and Antioxidant Properties in Mulberry Silkworm (*Bombyx mori* L.) Powder

**DOI:** 10.3390/foods9070871

**Published:** 2020-07-03

**Authors:** Artorn Anuduang, Yuet Ying Loo, Somchai Jomduang, Seng Joe Lim, Wan Aida Wan Mustapha

**Affiliations:** 1 Department of Food Sciences, Faculty of Science and Technology, Universiti Kebangsaan Malaysia, UKM Bangi 43600, Malaysia; a.anuduang@gmail.com (A.A.); joe@ukm.edu.my (S.J.L.); 2 Division of Food Science and Technology, Faculty of Agro-Industry, Chiang Mai University, Chiang Mai 50100, Thailand; somchai.j@cmu.ac.th; 3 Biosafe Holding Partnership Limited, 353 Moo 9, Tambol Sanklang, Sanpatong District, Chiang Mai 50120, Thailand

**Keywords:** silkworm, edible insects, thermal processing, antioxidant activities, silkworm powder

## Abstract

The mulberry silkworm (*Bombyx mori* L.) is a common edible insect in many countries. However, the impact of thermal processing, especially regarding Thai silkworm powder, is poorly known. We, therefore, determined the optimum time for treatment in hot water and subsequent drying temperatures in the production of silkworm powder. The silkworms exposed to 90 °C water for 0, 5, 10, 15, and 20 min showed values of Total Phenolic Compounds (TPCs), 2,2-Diphenyl-1-picrylhydrazyl free radical scavenging (DPPH) assay, 2,2′-Azino-bis(3-ethylbenzothiazoline-6-sulfonic acid (ABTS) assay, and Ferric Reducing Antioxidant Power (FRAP) assay that were significantly (*p* < 0.05) higher at the 5 min exposure time compared with the other times. The reduction of microorganisms based on log CFU/g counts was ≥3 log CFU/g (99%) at the 5 min treatment. To determine the optimum drying temperature, the silkworms exposed to 90 °C water for 5 min were subjected to a hot-air dryer at 80, 100, 120, and 140 °C. The TPC value was the highest (*p* < 0.05) at 80 °C. The silkworm powder possessed significantly (*p* < 0.05) higher DPPH, ABTS radical scavenging ability, and ferric ion reducing capability (FRAP assay) at 80 °C compared with other drying temperatures. This study indicates that shorter exposure times to hot water and a low drying temperature preserve the antioxidant activities. High antioxidant activities (in addition to its known protein and fat content) suggest that silkworms and silkworm powder can make a valuable contribution to human health.

## 1. Introduction

The custom of consuming insects as a food source (entomophagy) has been widely practiced by many people from all around the world for thousands of years [1]. Global population growth has led to the increase of food consumption, especially protein. Due to the high protein content, short life cycle, and rapid growth cycle, edible insects may serve as an alternative source of protein for humans, as suggested by Meyer-Rochow [2]. Food security is seen as one of the major global concerns, and therefore, the benefits of edible insect have been re-evaluated, as insects are rich in protein, fats, minerals, and essential vitamins, and contain low amount of carbohydrates. Moreover, insects are abundant and easily bred in large quantities [3]. The mulberry silkworm (*Bombyx mori* L.) is an edible insect that has been bred for a long time and can be produced throughout the year. Silkworms produce silk from plant proteins. In the sericulture industry, the after-silking silkworms are the main by-product and have become a popular food among the 194 species of edible insects in Thailand. In many Asian countries, silkworm has been utilized as food, medicine, and animal feed due to its nutritional profile [4]. Meyer-Rochow [5] mentioned that the larvae, pupae, and adults of *B. mori* could serve as medicine to treat wounds, sore throats, fever, bleeding, brain hemorrhage, hemorrhoids, and others. Besides phenolic compounds, silkworms also exhibit antioxidant activity [6]. Studies revealed that some pigments found on the yellow Nangnoi silk (Thai silk) are associated with the presence of carotenoids and flavonoids [7,8]. The phenolic compounds are well-known for antioxidant activity as well as some biological functions such as anti-hypertensive [9], antiviral [10], antioxidant [11], antibacterial, and anti-inflammatory activities [12]. Dutta et al. [13] reported that the antioxidant potential of the edible insect *Vespa affinis* L. could mediate its therapeutic activities in oxidative stress-associated health disorders. Dutta et al. [14] also suggested that the antioxidant potential of the edible insect, *Brachytrupes orientalis* extract in hydro-alcoholic (AEBO) has a significant role against cellular oxidative impairment.

Silkworm powder, also known as pury, is reported to have a high content of nutrients and tends to be an alternative functional food [15]. The yellowish silkworm powder was reported to possess a high content of nutrients such as protein, essential amino acids, polyunsaturated fatty acid, carbohydrates, vitamins, and minerals. Suk et al. [16] reported that the incorporation of silkworm powder in wheat flour noodles could reduce postprandial glucose response and act as a potential carbohydrate staple food for glycemic control. Park et al. [17] suggested that silkworm powder could be an alternative additive for meat products, as it improved the meat product’s physicochemical properties such as protein, fat, and ash contents. Biró et al. [18] concluded that the addition of silkworm powder in buckwheat pasta could increase its nutritional value.

A thermal process is usually employed in the food industry to reduce microbial activity and prolong the shelf life of silkworm products [19]. In addition, thermal processing of food has resulted in physical or chemical changes [20] to ensure the quality standards of food products. Baek et al. [21] stated that hot air drying and oven broiling methods gave the highest score in sensory evaluation in terms of the hardness, crispiness, and aroma in processed mealworm larvae. Meanwhile, Jensen et al. [22] reported that different processing methods were available to alter the taste, aroma, and texture of the insects. In this study, silkworm was processed into a yellowish powder by treatment in hot water, dried, and ground. Thermal processing may destroy some of the bioactive compounds and affect the antioxidant activity in food [23]. Norafida and Aminah [24] revealed that heat treatments (blanching, steaming, or boiling) changed the quality of food products such as texture, taste, color, and nutritional content. Hence, this study aimed to determine the optimal times for treatment in hot water and subsequent drying in the production of silkworm powder.

## 2. Materials and Methods

### 2.1. Sample Preparation

Fresh after-silking silkworms (FASSs) were collected from Natural Thai Golden Silk Ltd. (skincare manufacturing industry, Payao Province, Thailand). Fifth instar silkworms are used to produce silk sheets in the process of developing a skincare product. The silkworms are collected and disposed of as waste after silking. Thus, FASS was chosen in this study to transform the waste into a value-added product. The samples were transferred immediately to the laboratory in the Faculty of Agro-Industry, Chiang Mai University under cold conditions. The silkworm samples were then kept at −18 °C until further use.

### 2.2. Thermal Processing

#### 2.2.1. Hot Water Treatment (Pre-Treatment of Silkworms)

A total of 4 kg of frozen FASS was used in this study. FASSs were exposed to 90 °C water (ratio of 1:2) for 5, 10, 15, and 20 min. The treatment time started when the water temperature reached the desired temperature (90 °C). After each treatment time, the samples were blotted with tissue paper to remove the excess water on the surface of silkworms. The silkworm samples were further analyzed for their physicochemical properties and antioxidant activities. The experiments were carried out in triplicate.

#### 2.2.2. Drying Process

The silkworms that were exposed to 90 °C water for 5 min were then dried. The treated FASS was centrifuged at 1500× *g* rpm for 1 min to remove the excess water from the silkworm. FASS was dried in a hot-air dryer (Kluynamthai, Bangkok, Thailand) at four different temperatures (80, 100, 110, and 120 °C). The moisture content of the sample was measured every 30 min by recording the weight. Weight changes in the sample were used to calculate the moisture content of the sample. The drying process ended when less than 15% of moisture content was achieved for each drying temperature. The dried silkworms were ground into powder using a blender at 25,000 rpm (Nanotech, Beijing, China) and filtered using a 1.0 mm mesh sieve to obtain fine silkworm powder. The powder was then further analyzed for its physicochemical properties and antioxidant activities.

### 2.3. Physicochemical Properties

The color, size, and weight of the silkworm were determined in this study. The surface color of silkworms was measured using a Hunter Lab colorimeter (model Color Quest XE, Reston, VA, USA). The color scale gave the measurement of color (L*, a*, and b*) in units of approximate visual uniformity throughout the solid color. L* value determined lightness, where the score of 100 showed perfect white color and 0 for black color. Moreover, a* measured the redness when positive, whereas b* measured the yellowness when positive. The length and width of silkworms were measured by a Vernier caliper. The weight of the silkworm was recorded, and the average weight was calculated.

### 2.4. Proximate Analysis

Moisture content (method 934.01), ash (method 923.03), and total fat (method 991.36) were analyzed using the protocols of the Association of the Official Analytical Chemists (AOAC). Protein content (N × 6.25) was examined using the AOAC Kjeldahl method (984.13). Carbohydrate was obtained by the formula of 100 – (the sum of moisture, protein, fat, and ash).

### 2.5. Total Microorganism

The total microorganisms in the silkworm were determined using the aerobic plate count method, as described in the FDA’s Bacteriological Analytical Manual (Chapter 3) [25]. The silkworms (10 g) were pre-enriched in tryptic soy broth (90 mL; Merck, Darmstadt, Germany) in a 1:9 sample-to-broth ratio. The pre-enriched sample was diluted in broth by a 10-fold serial dilution (10^−2^, 10^−3^, and 10^−4^). Then, 1 mL of each dilution was pipetted and spread onto the plate count agar (PCA; Merck, Darmstadt, Germany). The agar plates were incubated at 37 °C for 24 h. The experiments were carried out in triplicate, and the results were expressed as log colony-forming units per gram (log CFU/g).

### 2.6. Antioxidant Assays

Extraction was performed before the antioxidant assays. Fresh silkworm, hot water-treated silkworm, and silkworm powder were weighed at 10 g each (dry weight equivalent) and mixed with 50 mL of 85% (*v*/*v*) ethanol (Merck, Darmstadt, Germany). The mixture of each sample was left at the ambient temperature for 1 h. The mixture was then filtered using Whatman^®^ Grade 4 filter paper.

### 2.7. Total Phenolic Compounds (TPCs)

Total phenolic compounds (TPCs) of the silkworms were determined by the Folin–Ciocalteu method with slight modification from Li et al. [26]. Approximately 100 µL of silkworm extract was mixed with 2 mL of 10% (*v*/*v*) Folin–Ciocalteu reagent (Merck, Darmstadt, Germany), and the mixture was left for 3 min at the ambient temperature. Then, 2.5 mL of 7.5% (*w*/*v*) sodium carbonate (Sigma, Darmstadt, Germany) was added to the mixture and incubated in the dark for 2 h. Absorbance was recorded using a spectrophotometer (BMG Labtech, Ortenberg, Germany) at 760 nm. Gallic acid was used as the standard for calibration. TPC was expressed in terms of µmol gallic acid equivalents per 1 g dry weight (DW) sample (µmol GAE/g of DW sample).

### 2.8. The 2,2-Diphenyl-1-picrylhydrazyl (DPPH) Free Radical Scavenging Assay

The determination of the scavenging activity of the silkworm extracts on the stable free radical DPPH was done as described in Phongthai et al. [27] with slight modification. The DPPH stock solution was prepared by dissolving 40 mg of DPPH (Sigma, Germany) in 100 mL methanol (Merck, Germany) to yield an absorbance of 0.70 ± 0.01 at 517 nm. Silkworm extract (100 µL) or blank were mixed with 0.1 mM of DPPH reagent (2.9 mL). The mixture was then left in the dark for 30 min at ambient temperature. The absorbance of the mixture was measured by spectrophotometer (Thermo Spectronic, Model Genesys 10UV-Scanning, CE, Wisconsin, WI, USA) at 517 nm. All samples and controls were done in triplicate. A standard curve was prepared using standard Trolox at concentrations of 0.01–0.40 mg/mL. Ethanol served as negative control (without samples). The values were expressed in terms of µmol equivalent of Trolox/dry basis (µmol TE/g DW).

### 2.9. The 2,2′-Azino-bis(3-ethylbenzothiazoline-6-sulfonic Acid (ABTS) Assay

Antioxidant activities of silkworm extract were determined using the method as described by Phongthai et al. [27] with slight modification. The stock solution was prepared by mixing 7 mM 2, 2-azinobis-3-ethyl-benzothiazoline-6-sulfonic acid (ABTS) reagent with 2.45 mM potassium persulfate (K_8_S_2_O_8_) at a ratio of 1:1. The mixture was left in the dark for 12 h at ambient temperature. After 12 h, the mixture was diluted with deionized water until the absorbance at 734 nm wavelength reached 0.70 ± 0.05 using the spectrophotometer. An aliquot of silkworm extract (150 µL) was mixed with 2.85 mL of ABTS radical cation stock solution before 2 h incubation in the dark. The absorbance of the mixture was measured at 734 nm. Ethanol served as negative control (without samples). The value was expressed in terms of µmol Trolox equivalent/g (µmol TE/g DW).

### 2.10. Ferric Reducing Antioxidant Power (FRAP) Assay

The antioxidant activity of silkworm extract was determined according to Phongthai et al. [27] with some modifications. The stock solution of FRAP reagent was prepared using 300 mM acetate buffer, pH 3.6 (3.1 g sodium acetate trihydrate and 16 mL glacial acid made up to 1:1 with distilled water), 10 mM TPTZ (2,4,6-tris [2-pyridyl]-s-triazine) in 40 mM HCl, and 20 mM FeCl_3_.6H_2_O in a ratio of 10:1:1. Approximately 0.8 mL of silkworm extract was added to 4 mL of FRAP reagent and left at ambient temperature for 10 min. The absorbance was measured at 595 nm using a spectrophotometer after 10 min. A standard calibration curve of ferrous sulphate (FeSO_4_) was plotted to estimate the activity capacity of samples. Ethanol served as negative control (without samples). The results were reported in terms of µmol Fe^2+^/g dry basis sample.

### 2.11. Statistical Analysis

The results were expressed as the mean ± standard deviation (SD) of three independent experiments. Statistical comparisons of the results were subjected to one-way ANOVA using SPSS version 20. The differences in antioxidant activity and TPC among the different exposure times to hot water at the *p* < 0.05 level of significance, and the differences in antioxidant activity and TPC among the different drying temperature at the *p* < 0.05 level of significance were analyzed by Duncan’s triplicates range test.

## 3. Results and Discussion

### 3.1. Physicochemical Properties of FASS

The physicochemical properties of FASS are shown in Table 1. FASS showed a high lightness with an L* value of 58.11 ± 0.63 and b* value of 24.45 ± 0.98, indicating that FASS in this study was yellowish. According to Taba and Gogoi [28], fifth instar silkworm exists in three forms, i.e., bluish-white, yellow, and bluish-green. FASS had a length of 33.19 ± 1.72 mm and a breadth of 6.97 ± 0.22 mm. Meanwhile, its weight was 1.04 ± 0.06 g. The size of FASS in this study was almost similar to the size of fifth instar larvae in the study of Gurjar et al. [29] with a length of 57.86 ± 7.06 mm and breadth of 7.15 ± 0.43 mm.

### 3.2. Proximate Analysis

The results of the proximate analysis are shown in Table 1. Protein has the highest percentage (66.66% of dry matter (DM)) among the components in silkworms. Karthick Raja et al. [30] reported that the protein content in dried silkworm ranged from 52 to 72%. The high content of protein in silkworm was due to the intake of mulberry leaves. Mulberry leaves are the only food source of the silkworm, and the leaves are reported to have high protein content [31].

Fats are identified as the second-highest percentage of components in silkworms. In this study, FASS contained 20.64% DM basis of fat content, which falls in the range of 18.9–36%, as reported by Kim et al. [32]. Meanwhile, Chieco et al. [33] mentioned that the silkworm contains essential fatty acids (EFA), mainly in the form of polyunsaturated fatty acids (PUFA). In addition, the silkworms also possess a higher n-3 to n-6 fatty acid ratio, which differs from other insects.

In this study, FASS exhibited high moisture content (75.83%), and this may have been due to the sole food source of the silkworm, the mulberry leaves [34]. The ash content of FASS was 1.32% on a dry matter basis. The ash content indicated the amount of minerals present in the insect. Omotoso [35] reported that the silkworm is rich in minerals such as sodium (Na), potassium (K), calcium (Ca), iron (Fe), magnesium (Mg), and zinc (Zn). In addition, the carbohydrate content was found to have a 2.59% DM basis in FASS. Carbohydrate is identified as the primary food source in silkworms.

### 3.3. Total Microorganisms

Table 1 shows that the microorganisms count of the silkworm was 6.54 log CFU/g. The incidence of a high amount of microorganisms is reasonable because the fresh silkworms were rich in nutrients with high moisture content. Li et al. [36] revealed that microorganisms could be found in the intestine of lepidopteran insects such as silkworm, which play essential roles in promoting growth and development.

### 3.4. Antioxidant Activities

A linear calibration curve of Trolox was obtained with good linearity of correlation coefficient (*R*^2^ = 0.979) and a linear equation of y = 185.3x + 18.97, which was used as positive control for DPPH scavenging activity. The value of the DPPH assay for FASS was 11.57 ± 0.80 µmol TE/g DW. In the FRAP assay, the antioxidant activity of FASS was determined by the ability of antioxidant compounds in silkworm to reduce Fe^3+^ to Fe^2+^ in the FRAP reagent. A linear calibration curve of ferrous sulphate (FeSO_4_) was obtained with good linearity of the correlation coefficient (*R*^2^ = 0.996) and a linear equation of y = 12.66x + 0.031, which was used as a positive control for the FRAP assay. The FRAP value of FASS was 54.28 ± 2.86 µmol Fe^2+^/g on a dry basis, indicating that the fresh silkworm exhibited a strong ferric ion reducing capacity. A linear calibration curve of Trolox was obtained with good linearity of correlation coefficient (*R*^2^ = 0.982) and a linear equation of y = 771.1x + 9.971, which was used as a positive control for ABTS scavenging activity. The value of the ABTS assay of FASS in this study was 32.75 ± 4.57 µmol TE/g DW. A higher ABTS radical scavenging capability was observed in FASS compared with DPPH measurement. This could be due to the high protein content in fresh silkworm, as reported by Zheng et al. [37], whereby the ABTS assay was more sensitive in evaluating the radical scavenging activities of amino acids and peptides compared with the DPPH assay.

### 3.5. Effect of Hot Water Treatment

**Figure 1:**
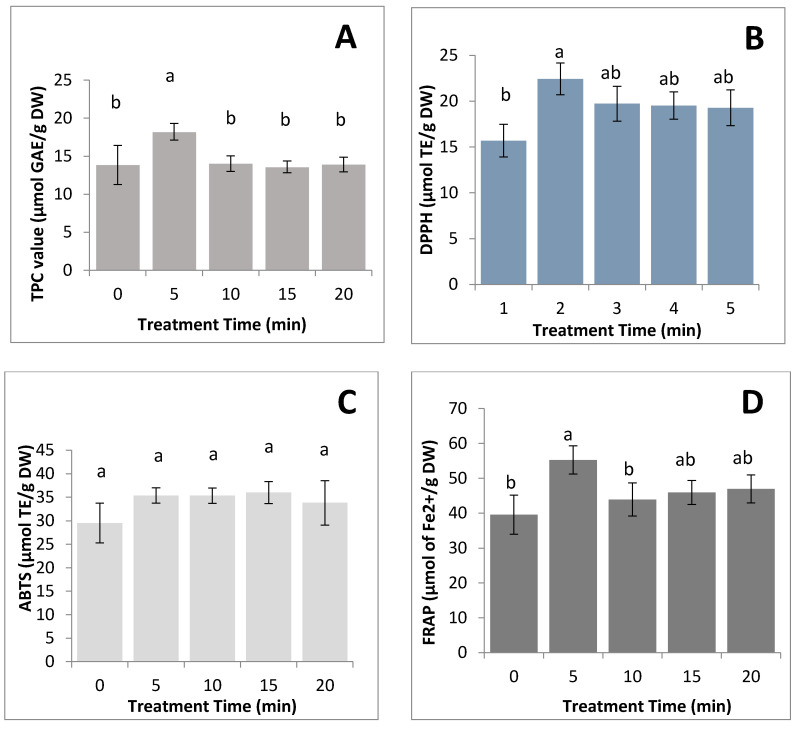
Effect of different treatment time of hot water on antioxidant activity in silkworms. (**A**) Total phenolic compound (TPC), (**B**) DPPH free radical scavenging assay, (**C**) ABTS assay, and (**D**) ferric reducing antioxidant power (FRAP). The results were expressed as mean ± SD of triplicate measurements. The different letter on the bar indicates significantly difference among treatment (*p* < 0.05).

FASS was exposed to 90 °C water before the process of producing silkworm powder. The exposure of FASS to hot water was to kill them thoroughly and for sterilization purposes. As shown in Figure 1A, the TPC of FASS (18.21 ± 1.31 µmol GAE/g DW) was significantly (*p* < 0.05) higher following 5 min of exposure compared with the other time points. The values of DPPH free radical scavenging assay (22.43 ± 2.85 µmol TE/g DW), ABTS assay (35.37 ± 2.99 µmol TE/g DW), and FRAP assay (55.26 ± 4.05 µmol of Fe^2+^/g DW) were significantly (*p* < 0.05) higher following 5 min exposure compared with the other time points (Figure 1B–D). Therefore, the samples exposed to 90 °C water for 5 min were used for subsequent drying treatment.

The TPC, DPPH free radical scavenging assay, and FRAP assay of FASS showed a significant reduction (*p* < 0.05) when the exposure time was extended to 10, 15, and 20 min. The reduction of TPC indicated the loss of antioxidant compound, and this was due to the large surface area of FASS in contact with water [38]. In addition, cooking in the boiling water had an adverse effect on the polyphenol level, as it could diffuse into the boiling water, resulting in the loss of phenolic compounds [39]. On the other hand, it was interesting to note that TPC and antioxidant activity of FASS were significantly (*p* < 0.05) increased at the 5 min exposure time and gradually decreased after exposure to hot water for 10, 15, and 20 min. The hot water treatment caused the destruction of cell and sub-cellular components in the silkworms, resulting in the release of potent radical-scavenging antioxidants [40]. Furthermore, a study suggested that the thermal inactivation of oxidative enzymes during the heating process led to the suppressed oxidation by antioxidants [39].

Table 2 shows that the total microorganism load at 0 min of exposure time was 6.69 log CFU/g. Following exposure for 5 min, the microorganism count was 1.30 log CFU/g, while the total microorganism count was less than 1.0 log CFU/g for exposure times of 10, 15, and 20 min. It was observed that the reduction in the number of CFU/g was ≥3 log CFU/g (99%). The whole body of the silkworm, including the intestine, was used in the production of silkworm powder. The intestine of silkworm contains a high amount of microorganisms, which affect the quality and shelf life of silkworm powder [41]. Hence, a pre-treatment of the fresh silkworm is an essential step before the production of silkworm powder.

### 3.6. Effect of Drying Temperature

The silkworms exposed to 90 °C water for 5 min were subjected to a hot air dryer. Table 3 shows the effect of different drying temperatures on the physicochemical properties and antioxidant activities of silkworms. For the color measurement of silkworm powder, L* and b* values were significantly (*p* < 0.05) decreased when the drying temperature increased. The color fading in the silkworm powder may have been due to the sensitivity of the silkworm color pigment to high temperature [42]. In addition, Dorouzi et al. [43] reported the formation of browning pigments by the non-enzymatic browning or Maillard reactions at high drying temperature, leading to a decrease in lightness. The moisture content of the silkworm powder was measured until the final moisture content was less than 15%, as the growth of microorganism could be prevented when the moisture content was reduced to less than 15% [44,45].

The TPC of silkworm powder was significantly (*p* < 0.05) decreased at 100 °C and increased at 120 and 140 °C. This was due to the release of potent radical-scavenging antioxidants, as reported by Kao et al. [40]. However, the results showed that the TPC of silkworm powder was significantly higher (51.81 ± 0.84 µmol GAE/g DW) at a low drying temperature (80 °C). The DPPH free radical scavenging assay showed a significant (*p* < 0.05) decease as the drying temperature increased and the highest value of DPPH free radical scavenging (69.09 ± 0.58 µmol TE/g DW) was obtained at 80 °C. The results of ABTS and FRAP assays showed a significant (*p* < 0.05) reduction in silkworm powder as the drying temperature increased. In the ABTS assay, silkworm powder possessed the highest ABTS radical scavenging capability (69.27 ± 2.85 µmol TE/g DW) of antioxidant at 80 °C. The FRAP assay of silkworm powder showed a significantly (*p* < 0.05) higher value at 80 °C compared with the other drying temperatures. The decreased antioxidant activity upon exposure to high temperature was associated with the degradation of antioxidant compounds [46]. Furthermore, the decrease in antioxidant activity in silkworm powder was due to the formation of pro-oxidants by the Maillard reaction when exposed to high temperature [47].

The presence of antioxidant activity in fifth instar silkworms is probably because of the production of sericin. Silkworms produce large quantity of sericin at the fifth instar stage larvae. Sericin was reported to have high antioxidant properties [48]. The high consumption of mulberry leaves during late larval stage contributes to the antioxidant activity, as mulberry leaves have been identified to contain large quantities of phenolic compounds such as flavonoid and its derivatives [49]. Thermal processing, especially water heat treatment, may cause the loss of phenolic compounds, as reports showed that phenolic are highly soluble in water. Phenolics may be lost by leaching. The high temperature may destroy the cell wall and other subcellular compartments, thus facilitates the release of the compound into the water [50]. The reduction of antioxidant compounds may due to certain factors such as temperature, air velocity, heat exposure time, and the content of antioxidant compound [51].

## 4. Conclusions

Silkworm and silkworm powder contain antioxidant activities. The results showed that thermal processing could affect the antioxidant activity and some physicochemical properties during the production of silkworm powder. Shorter exposure time to hot water and a lower drying temperature should be applied to prevent the loss of bioactive compounds in silkworm powder. Silkworm powder could be a potential protein source for animal products, and the application may lead to valuable findings in various fields such as pharmaceutical and cosmetic industries.

## Figures and Tables

**Table 1:** Physicochemical properties, total microorganism count and antioxidant activity of fresh Thai silkworm after silking (FASS).

Properties	Value
**Physicochemical Properties**	
Color L*	58.11 ± 0.63
a*	1.14 ± 0.99
b*	24.45 ± 0.98
Size (mm) length	33.19 ± 1.72
Width	6.97 ± 0.22
Weight (g)	1.04 ± 0.06
**Proximate Analysis**	
Moisture content (%)	75.83 ± 0.63
Protein (% DM basis)	66.66 ± 1.23
Fat (% DM basis)	20.64 ± 2.24
Ash (% DM basis)	1.32 ± 0.13
Carbohydrate (% DM basis)	2.59 ± 0.43
**Total Microorganism** (log CFU/g)	6.54 ± 0.13
**Antioxidant Activities**	
DPPH (µmol TE/g DW)	11.57 ± 0.80
ABTS (µmol TE/g DW)	32.75 ± 4.57
FRAP (µmol of Fe^2+^/g DW)	54.28 ± 2.86

**Table 2:** Effect of water heat treatment time on total microorganism load in fresh after silking silkworms.

Treatment Time (min)	Total Microorganisms Load (log CFU/g)
0	6.69 ± 0.39
5	1.30 ± 0.38
10	<1
15	<1
20	<1

**Table 3:** Chemical properties and antioxidant activities of dried silkworms in a hot-air oven at different drying temperatures.

Properties	Drying Temperature (°C)			
	80	100	120	140
Color L*	47.07 ^a^ ± 0.27	44.80 ^c^ ± 0.24	46.13 ^b^ ± 0.40	44.42 ^c^ ± 0.44
a*	4.68 ^c^ ± 0.10	5.83 ^b^ ± 0.24	6.61 ^a^ ± 0.22	6.67 ^a^ ± 0.08
b*	17.01 ^a^ ± 0.38	14.36 ^c^ ± 0.68	15.96 ^b^ ± 0.50	13.75 ^c^ ± 0.28
Moisture content (%)	8.08 ^a^ ± 0.37	4.30 ^b^ ± 0.30	2.20 ^c^ ± 0.59	0.70 ^d^ ± 0.18
Total phenolic compounds (µmol GAE/g DW)	51.81 ^c^ ± 0.84	47.12 ^a^ ± 0.77	48.96 ^ab^ ± 1.53	50.29 ^bc^ ± 2.12
**Antioxidant Activities**				
DPPH (µmol TE/g DW)	69.09 ^b^ ± 0.58	68.59 ^ab^ ± 0.31	68.71 ^b^ ± 0.15	67.94 ^a^ ± 0.37
ABTS (µmol TE/g DW)	69.27 ^b^ ± 2.85	68.90 ^b^ ± 0.56	68.29 ^b^ ± 0.87	57.63 ^a^ ± 0.91
FRAP (µmol of Fe^2+^/g DW)	46.73 ^b^ ± 4.54	43.02 ^ab^± 2.23	41.71 ^ab^ ± 2.61	39.89 ^a^ ± 2.17

The results were expressed as mean ± SD (*n* = 3) of triplicate measurements. Mean values with the different superscript lowercase letter are significantly different (*p* < 0.05).
